# Extensible Multiplex Real-time PCR of MicroRNA Using Microparticles

**DOI:** 10.1038/srep22975

**Published:** 2016-03-11

**Authors:** Seungwon Jung, Junsun Kim, Dong Jin Lee, Eun Hae Oh, Hwasup Lim, Kwang Pyo Kim, Nakwon Choi, Tae Song Kim, Sang Kyung Kim

**Affiliations:** 1 Center for BioMicrosystems, Brain Science Institute, Korea Institute of Science and Technology (KIST), Seoul, Korea; 2 Department of Chemical & Biological Engineering, Korea University, Seoul, Korea; 3 Center for Imaging Media Research, Robot & Media Institute, KIST, Seoul, Korea; 4 Department of Applied Chemistry, The Institute of Natural Science, College of Applied Science, Kyung Hee University, Seoul, Korea; 5 Department of Biomedical Engineering, Korea University of Science and Technology (UST), Daejeon, Korea

## Abstract

Multiplex quantitative real-time PCR (qPCR), which measures multiple DNAs in a given sample, has received significant attention as a mean of verifying the rapidly increasing genetic targets of interest in single phenotype. Here we suggest a readily extensible qPCR for the expression analysis of multiple microRNA (miRNA) targets using microparticles of primer-immobilized networks as discrete reactors. Individual particles, 200~500 μm in diameter, are identified by two-dimensional codes engraved into the particles and the non-fluorescent encoding allows high-fidelity acquisition of signal in real-time PCR. During the course of PCR, the amplicons accumulate in the volume of the particles with high reliability and amplification efficiency over 95%. In a quick assay comprising of tens of particles holding different primers, each particle brings the independent real-time amplification curve representing the quantitative information of each target. Limited amount of sample was analyzed simultaneously in single chamber through this highly multiplexed qPCR; 10 kinds of miRNAs from purified extracellular vesicles (EVs).

As researchers seek out and unveil the divergent roles of nucleic acids, the quantitative as well as qualitative analyses of nucleic acids become increasingly important^1^. In particular, the quantitative profile of miRNAs has drawn much attention as miRNAs have recently been recognized as novel diagnostic parameters for significant diseases such as inherited diseases, cancers, heart diseases, infection, alcoholism, and obesity. qPCR is considered to be the gold standard in miRNA expression analysis because it is a well-characterized methodology with a wide dynamic range and low limit of detection compared to northern blot or microarray^2^. Until recently, however, the number of multiplicity in common qPCR has been limited to a maximum of six due to the restricted number of color channels^3^.

To overcome the limited multiplicity of qPCR, new methods have been reported and have been rapidly commercialized encompassing more than tens of miRNAs at a time. In the nCounter system from NanoString, probe pairs with color codes hybridize to targets *in situ* and hundreds of miRNAs have been detected via single ligation reaction^4^. However, this amplification-free method often misses less abundant targets and then it is compensated with reverse transcription (RT)-qPCR subsequently^5^. Similarly, another hybridization-based approach has brought in the ligation of probes to miRNAs but focuses on the sensitivity lowering the limit of detection^6^,^7^. The probes intensify the signal through enzymatic amplification, taking the trade off in quantitative resolution. A microwell-array qPCR, in contrast, can parallelly integrate tens of the conventional real-time PCR with the aid of high-end microfluidic chips and precise mechanical controllers to manipulate multiple primers and samples in nL range^8^.

Here, we present a multiplex qPCR for miRNA profiling equipped with novel particles, lithographically encoded microparticles of primer-immobilized networks (LEM-PIN). PIN, composed of polyethylene glycol (PEG), is highly porous and hydrophilic so that the amplification reaction in the particles is as efficient as in an aqueous media. Each LEM-PIN of a specific primer is identified by an engraved pattern, which has a coding capacity of much greater than the number of human miRNAs. Thus, the addition of a relevant LEM-PIN readily expands the target of analysis to the limit allowed by the space available. No other qPCR platform has indicated multiplexing capacity to thousand-plex in conjunction with such simple and flexible workflow. Moreover, the lithographic encoding, absent from fluorescence, brings a robust signal acquisition in real-time PCR where fluorescent change must be monitored between every cycle.

## Results

### Facile production and identification of particles

**Figure 1:**
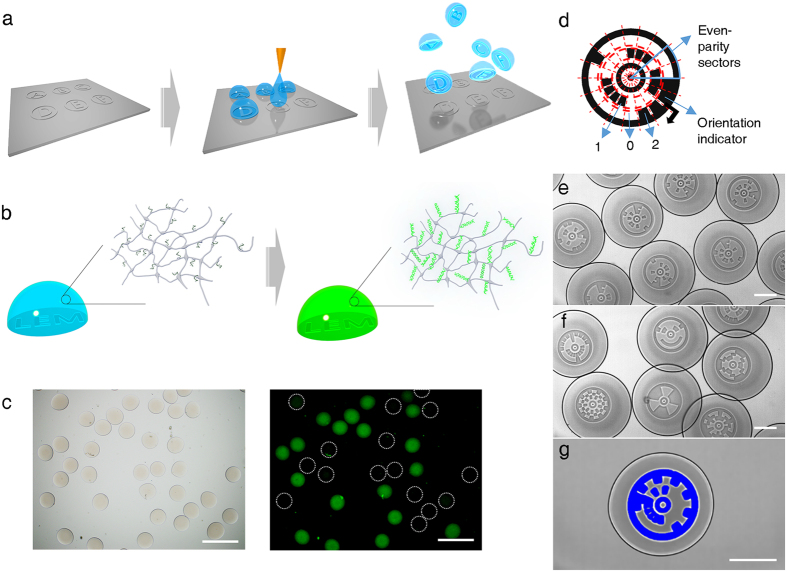
Lithographically Encoded Microparticle-Primer Immobilized Network (LEM-PIN). (**a**) Schematic of LEM-PIN fabrication composed of jetting the solution on pre-patterned PDMS. (**b**) Accumulation of PCR amplicons and its intercalating fluorescence inside LEM-PIN. (**c**) Bright-field and fluorescence images after multiplex PIN PCR. Scale bars are 500 μm. (**d**) Ringcode symbol structure. This consists of 16 sectors with a ternary code on each sector with encoding capacity of 3^12^. (**e**) Various ringcodes. (**f**) Ringcode-based patterns. (**g)** Post-processing of the code images for enhancement of decoding. Scale bars for e, f, and g are 200 μm.

The photo-curable hydrogel is widely used for various tissue-fixing or particle-based bioassay due to its facile porosity control and high conformity with aqueous biochemical reactions^9^,^10^,^11^. We formed PIN composed largely of polyethylene glycol (PEG) as its well-known acrylate chemistry allows for an abundant incorporation of primers throughout the volume of the particles^6^,^7^,^12^,^13^,^14^. A drop-casting process was developed to integrate the identification codes into the PIN, resulting in the LEM-PINs for particle-based qPCR as shown in Figs 1a,b and S1. The aqueous solution of pre-polymer, by the volume of several nl, was dropped over pattern-engraved spots on a hydrophobic substrate. The array of micro-drops was cured by brief UV irradiation and then, solidified. The released particles from the substrate possessed their printed codes; 5 μm in height and readable in bright field mode. Their well-defined contrast is induced by the light scattering which occurs along the contour of the patterns at the bottom of the LEM-PINs. The particles were shaped hemispherically because they were originally sessile drops spotted on the hydrophobic surface. In most cases, the flat bottom of LEM-PINs was placed at an observational focal plane so as to facilitate decoding in the bright field mode (Fig. S2). The volume of LEM-PINs could be adjusted from 3 nl to 1 μl with a standard deviation of less than 10% as shown in Fig. S3. The LEM-PINs could be produced precisely with a high throughput of about 15,000 particles/hr per channel, guaranteeing the particles’ uniformity requisite for quantitative analysis.

For the systematic encoding of the particles, circular ringcodes were designed as inspired by the shot codes^15^ composed of two concentric rings with 16 sectors (Fig. 1d). Four of the sectors were occupied by starting, directional and error codes and the 12 remaining sectors encoded a ternary identifier. The resulting encoding capacity was 3 12 which exceed the required multiplicity amount for most of applications. Figure 1e,f detail several ringcodes of different patterns which includes special structures illustrating the freedom of the patterns. In addition to the strong readability of the codes under simple microscopes, post-processing on the images could be used to enhance contrast for robust automatic decoding as in Fig. 1g. Figure S4 showed the example of automatic decoding using the Hough transform and GrabCut algorithm^16^,^17^. The outer circle enclosing the ringcode was detected and then the code pattern and the dark region were segmented from the detected circle. Once the digitized pattern was segmented out from the image, it was easily decoded as ternary code.

### Real-time PCR in PIN particles

The quality of the PCR in the particles is dependent on the characteristics of the PIN with regards to the size, density and distribution of nanopores as well as to the activity of the primers. Mass transfer through the network was optimized by controlling the composition of the PEGDA, porogen and UV irradiation dosage to ensure that nanopores were over 16 nm in width and that more than 80% of the volume was occupied by an aqueous solution where the dNTP, polymerase, target template, and amplicons were dissolved and swimming through^12^,^18^. A primer concentration of over 5 fmol/nl was sufficient for the rapid amplification used in this study and 100 fmol/nl primers were maintained active in the following experiments (Table S1). As a result, a fixed primer and the other supplied reagents participated in the amplification reaction as in the homogenous solution.

**Figure 2:**
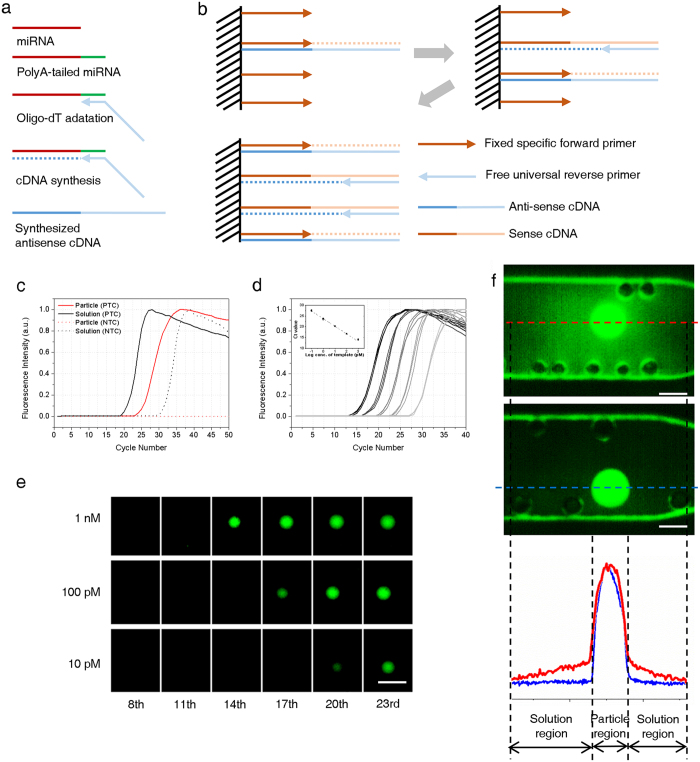
Design and performance of qPCR with LEM-PINs. (**a**) Reverse transcription of miRNA based on polyA tailing method. (**b**) Detailed scheme for accumulation of amplicons in the LEM-PIN during PCR. (**c**) Comparison between the conventional and LEM-PIN qPCRs. The Ct value of the particle qPCR for PTC was increased by 3.97 with standard deviation of 0.28 compared to that of the conventional qPCR. Interestingly, particle qPCR showed no signals for NTC even up to 50^th^ cycle while the conventional qPCR recorded a Ct value for NTC around the 30^th^ cycle. (**d**) qPCR results for a 10-fold serial dilution of miR-9-3p. The graphs kept the uniform distances among them which are about 3.3 corresponding to the 10-fold difference in template concentration. Inset shows the standard curve drawn based on serial dilution results, which indicate the reliability of the particle qPCR recorded a PCR efficiency of more than 95%. (**e**) Snapshots of particle qPCR with the concentration variation of templates. The particles became fluorescent in sequence according to the concentration of template. scalebar is 1 mm. (**f**) Rinsing effect of the particle. Insufficiently rinsed particle (upper) showed dim fluorescence around it while perfectly rinsed one has clear contrast from the background. This could lead to the influence to the other surrounding particles when multiplexing. Scalebar is 500 μm.

To expedite the miRNA qPCR in the LEM-PINs, we adjusted an assay on the poly(A)-tailing-reverse transcription (RT) favored for multiplex miRNA analysis^19^ (Fig. 2a). In this procedure, the complementary DNAs (cDNAs) were generated en masse through RT with one universal RT primer binding to the common polyadenylated tails of the miRNAs. For the PCR of a specific cDNA, a specific forward primer and a universal reverse primer were needed. Thus, the LEM-PIN for each miRNA contained only the specific forward primer needed while the universal reverse primer was provided from the master mix solution. Accordingly, the amplicons accumulated at the site of immobilized specific primers; thus, the fluorescence from the SYBR green I accumulated in the particles (Fig. 2b).

While PCR process keeps most of amplicons within the particle, the some antisense cDNAs which diffused into the surrounding solution were excluded from the amplification and remained non-fluorescent to themselves. This local confinement of fluorescence rendered the individual particles virtually isolated even though the mass transfer through the particles and solution was free from obstruction. In real assays, a key factor for signal isolation is the complete removal of unbound primers before PCR. In the presence of the residual unbound primers, dim fluorescence of surrounding solution appeared from out-diffused specific primers, which affected the signal of neighboring particles. (Figs 2f and S5) The authentic signal isolation between particles kept the assay simple, free from the need for oil encapsulation or leak-proof control of micro-wells which would be indispensable in microwell-array qPCR.

An additional merit of the LEM-PINs in qPCR was manifested through the comparative analysis with the conventional qPCR where all primers, targets and other reagents worked in homogeneous phase. The signal from the no-template control (NTC) was significantly suppressed in each qPCR with LEM-PINs (Fig. 2c). Without the relevant cDNAs, no fluorescence from the LEM-PIN appeared until after the 50th cycle, whereas in the conventional qPCR, the Ct value for NTC was approximately 30 with the identical no-template samples and primers. Based on this observation, we assume that primer-dimers in the LEM-PIN decreased significantly by virtue of the fixed specific primers which caused less random binding with the other primers. The constraint on primers seemed more preferential for the qPCR of rare targets where the Ct value was over 30 (Fig. S6).

Another important aspect in miRNA expression analysis is the quantitative resolution. The average signal of three particles in each halved concentrations clearly showed the fine quantitative resolution of the assays as shown in Fig. S7. The uniformity of LEM-PINs recorded precise Ct values in repeating experiments (Table S2 and Fig. S8). The high efficiency of PCR also sharpens the quantitative resolution of the assays (Fig. 2d). It was calculated to be 95.3% which is comparable to the ideal amplification efficiency of PCR. While deducing the efficiency, a series of 10-fold diluted target cDNAs were quantitated to 100 fM (equivalent to 1.6 amol of miRNA), covering a five-log range of concentration to 10 nM of the miRNA.

### Multiplex qPCR with LEM-PINs

The PIN qPCR can be paired with various encoding methods depending on the level of multiplicity. However, the intrinsic absence of fluorescent interference in the LEM-PINs renders them particularly suitable for real time PCR in contrast to fluorescent encoded particles^6^,^7^,^12^,^13^,^14^,^20^,^21^,^22^,^23^. Accordingly, multiplex qPCR was carried out with several kinds of LEM-PINs containing each specific primer following the decoding of the particles. In the case of a single-target, miR-9-3p introduction, only the particle of the relevant primer indicated a Ct value, 19.12, while the other particles for miR-16-5p or U6 snRNA remained non-fluorescent at the end of the assay (Fig. S9). This indicates, again, the occurrence of a clear signal isolation among the LEM-PINs and the suppression of non-specific signals in the particles compared to those of the conventional qPCR.

**Figure 3:**
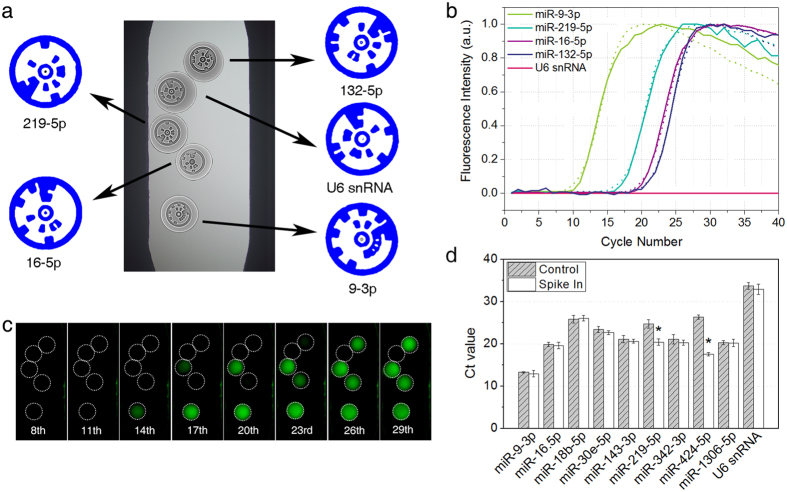
Multiplex qPCR with LEM-PINs. (**a)** Bright-field image and detailed code images of LEM-PIN array containing different primers in the channel. Each particle has its own code indicating the primer information. (**b**) Multiplex qPCR graphs with five different LEM-PINs. Solid and dotted lines represent the multiplex and singleplex assay, respectively. The graphs from multiplex assay nearly overlapped those from singleplex assay, which indicates no interference among the amplicons during amplification (**c**) Snapshots of the multiplex qPCR at cycles. (**d**) For practical use, the miRNAs of EV from K562 cell line were analyzed by our protocol. As comparing between control and spike-in samples, only spiked-in targets (asterisks) showed downshift in Ct value which means no significant influence among multiple targets. All the scalebars indicate 500 μm.

When miR-9-3p and miR-219-5p were mixed in a sample at similar concentrations, their Ct values from relative LEM-PINs were 18.39 and 19.17, respectively, which were consistent with those of the singleplex (Fig. S9). The performance of individual qPCR was sustained despite the number of targets increasing to five (miR-9-3p, miR-219-5p, miR-132-5p, miR-16-5p, and U6 snRNA) with corresponding LEM-PINs. Each of the multiple LEM-PINs recorded Ct values identical to those of the singleplex analysis when the concentration ranged from 10 pM to 10 nM. Figure 3b verified the stability of the individual qPCR for multiple targets without biased amplification^24^. In this light, the multiplicity of LEM-PIN qPCR, at the coding capacity over 10^5^, seems only limited by the size of particles. With LEM-PINs of 100 μm–diameter, 100 particles can be analyzed within a 1 mm^2^ well. Those particles also proved to be stable even after months of storage (Fig. S10). Thus, the assay could be expanded on demand by selecting target-specific particles. Following the completion of qPCR, multiplex melting curve analysis confirmed the single type of amplicons in each particle (Fig. S11).

In order to extend the use of LEM-PIN in practice, ten kinds of miRNAs in extracellular vesicles from human chronic myelogenous leukemia cell line (K562) were analyzed in single qPCR. After spike-in of two arbitrary miRNAs, miR-219-5p and miR-424-5p, to the sample, the quantitative profile was kept stable for the other miRNAs as shown in Figs 3d and S12. It proved the efficiency of LEM-PIN qPCR in multi-target analysis of real samples. The particle-based qPCR facilitated expression profiling of miRNAs from limited physiological specimens.

## Discussion

LEM-PINs were produced from a PEG/primer mixed solution with high precision and flexible size control. A number of patterns for identification can be engraved in the particles from the micro-fabricated PDMS molds. The two dimensional codes on the particle can be read clearly in the bright field mode and the fluorescence signal from the particles is monitored on the spot without interference, which enables easy workflow of real time PCR. Each particle showed an accumulation of fluorescence during PCR without diffusion of the signal to the surrounding solution or to other particles. This particle qPCR provided superior specificity and quantitative resolution compared to the conventional qPCR. Among other things, the number of targets detectable in single well is easily extended by virtue of lithographically encoded patterns. Multiple LEM-PINs simultaneously analyzed cDNAs which originated from target miRNAs with a wide concentration range of 10^3^ in a 1-μl sample. Present assay with LEM-PINs can be transformed into a multiplex qPCR of other genetic contents such as RNA and DNA in the future. This approach offers substantial opportunities for the realization of extensive genetic profiling on limited clinical specimens such as tumor tissue or extracellular body fluids.

## Methods

### Preparation of pre-patterned PDMS and LEMs

A Polydimethysiloxane (PDMS) mold was prepared from a replica on a Silicon master fabricated through photolithography using SU-8 2005 (MicroChem, Newton, MA) of 5 μm in height. 20% v/v Poly(ethylene glycol) diacrylate (PEG-DA, Sigma-Aldrich, Mn = 700), 40% v/v Poly(ethylene glycol) (PEG, Sigma-Aldrich, Mn = 600), 35% v/v 3X Tris-EDTA buffer (TE, Sigma-Aldrich) with 0.15% Tween-20 (Sigma-Aldrich) and 5% v/v Darocur 1173 (Sigma-Aldrich) were mixed as a pre-polymer solution. The solution for the PIN was finalized by mixing the pre-polymer solution and 1 mM acrydited DNA as a forward primer with a volume ratio of 9:1. PEG600 was used as a porogen maintaining pores for facile mass transfer during the amplification reaction. The LEM-PINs were produced by dropping pre-polymer solution on the pre-patterned PDMS using a jetting system (Arrayer 2000, Advanced Technology Inc., Korea) followed by a UV cross-linking of 35 mJ/cm^2^. The pre-polymer drop array was automatically generated after the manual recognition of three aligning marks on the PDMS. In order to control the volume of the drops, the opening time was tuned from 150 μsec to 500 μsec and the volumes of 100 drops were averaged to obtain a mean value. Cured LEM-PINs were easily released from the PDMS through mild agitation and they had the codes replicated on the bottom of the microparticles. The preparation of the LEM-PINs was completed through a rinsing process with a 1× TE buffer of 0.05% Tween-20 to remove the porogens and the unbound primers. The completed particles were stored at 4 °C soaked in a 1× TE buffer with 0.05% Tween-20.

### Decoding the codes on LEM-PINs

After filling the LEM-PINs and the PCR master mix to the PCR chamber, the codes were read in a bright-field mode of microscope. The codes can be read under either transmission (Observer.A1, Zeiss, Germany) or reflection (Axioplan2, Zeiss, Germany) microscopes with CCD cameras, OptiMOS (QImaging, Canada) and Retiga6000 (QImaging, Canada), respectively. The starting point and decoding direction were determined by recognizing the starting pattern consisting of two sectors. The codes can be interpreted as ternary codes. Each sector had inner and outer parts. The filled areas of these two parts represented one and two, respectively, while non-filled area represented zero. The last two sectors were used as even-parity sectors for the error detection of the codes. The total number of 1(3) and 2(3) of the code should be even as including even-parity sectors in order to be accepted as an authentic code.

### Reverse transcription of miRNA

Each standard miRNA mimic was purchased from Sigma-Aldrich. One microgram of miRNA was reverse-transcribed into a first-strand cDNA using the QuantiMir RT Kit (System Biosciences, Mountain View, CA), which transfers a miRNA into a cDNA of proper length for qPCR. Initially, the miRNA solution was incubated with a PolyA polymerase at 37 °C for 30 min so as to add a polyadenyl group at the 3’ end of the miRNA. After adding the oligo dT adaptor, it was heated to 60 °C for 5 min and then cooled down to room temperature for 2 min in order to help the adaptor bind to the polyadenylated miRNA. Finally, cDNA was synthesized by mixing Reverse Transcriptase and dNTP, consequently incubating the mixture at 42 °C for 60 min and finally heating it to 95 °C for 10 min to deactivate the Reverse Transcriptase. Resulting solution of cDNAs was stored at −20 °C until PCR.

### Real-time quantitative PCR with LEM-PINs

Singleplex, multiplex particle-based qPCRs and conventional solution qPCR were carried out using the UltraFast LabChip Real-time PCR G2-3 System (Nanobiosys, Seoul, Korea). In the conventional solution qPCR as a reference for comparison, 1 μl template cDNA was mixed with 8 μl 2× SYBR Green master mix (Nanobiosys), 1.6 μl of each of 1 μM forward and reverse primers, and 3.8 μl deionized water in a final volume of 16 μl which would be enough to fill the PCR chamber. For qPCR with LEM-PINs, the same protocol as above was used except that the forward primers were absent from the PCR master mix. A two-step amplification program was used which included 3 sec at 95 °C (denaturation) and 11 sec at 60 °C (annealing) for 40–50 cycles after the pre-denaturation step at 95 °C for 8 sec. The fluorescent images were saved at each cycle. Their intensities were recorded and all the data were normalized by the maximum intensities.

### Cell culture and isolation of EVs from cell media

Fresh cell media was harvested from K562 cell line which was cultured in RPMI 1640 media supplemented with 10% fetal bovine serum (FBS) and 1% penicillin/streptomycin at 37 °C under 5% CO_2_ followed by culture without FBS for the last 24 h. The cell media were then centrifuged at 800 g for 30 min to remove cell debris. The 140-ml supernatant containing the cell-free cell media was transferred to a fresh tube and held on ice till use.

### miRNA extraction from EVs

To purify EVs, sequential ultracentrifugation on a sucrose cushion and OptiPrep (50% iodixanol, Axis-Shield PoC AS, Oslos, Norway) density gradient were performed repeatedly. Firstly, purified media was loaded onto 0.8 and 2.7 M sucrose cushions in 20 mM HEPES/150 mM NaCl buffer (pH 7.2) and subsequently ultracentrifuged at 100,000 g for 2 h at 4 °C. [1,2] Consecutively, fractions containing EVs were added onto 0.8 and 2.7 M sucrose cushions after dilution in sucrose dilution buffer (20 mM HEPES/150 mM NaCl, pH 7.2), before ultra-centrifugation at 200,000 g at 4 °C for 70 min. The EV fractions on upper side of the 0.8 M and 2.7 M sucrose cushion were harvested to apply for step-density gradient ultracentrifugation (2.5 ml of 5% OptiPrep, 3 ml of 20% OptiPrep and 4.8 ml of 30% OptiPrep). Ten fractions of equal volume were collected from the top of the tube. The third fraction (1.4 ml) containing EVs was transferred to 9 mL PBS followed by ultracentrifugation at 100,000 g for 1 h at 4 °C to collect EV pellet for miRNA extraction. The miRNAs were extracted directly from the purified EVs by heating to 100 °C for 10 min and gathering supernatant after centrifugation to maintain fresh miRNA state.

### Reverse-transcription of controlled and spiked-in samples

The reverse-transcription of miRNAs from EVs was carried out using QuantiMir RT Kit (System Biosciences, Mountain View, CA) following manufacturer’s instruction right after miRNA extraction from EVs. For spike-in, 2 μl standard miRNAs (0.2 ng/μl miR-219-5p and 2 ng/μl miR-424-5p) were added to 3 μl miRNA pool obtained from EVs while 2 μl DI water was added to them instead of standard miRNAs for control.

## Additional Information

**How to cite this article**: Jung, S. *et al.* Extensible Multiplex Real-time PCR of MicroRNA Using Microparticles. *Sci. Rep.* **6**, 22975; doi: 10.1038/srep22975 (2016).

## Supplementary Material

Supplementary Video

Supplementary Information
