# Supplementary material for: Extensible Multiplex Real-time PCR of MicroRNA Using Microparticles
Source: Sci Rep. 2016 Mar 11;6:22975. doi: 10.1038/srep22975 (PMC4786821; doi:10.1038/srep22975)
Supplement: Supplementary Information [file srep22975-s2.pdf]

Supplementary information

Extensible Multiplex Real-time PCR of MicroRNA Using Microparticles

Seungwon Jung[1], Junsun Kim[1,2], Dong Jin Lee[1], Eun Hae Oh[1], Hwasup Lim[3], Kwang Pyo Kim[4], Nakwon Choi[1], Tae Song Kim[1], and Sang Kyung Kim[1]*

[1]Center for Biomicrosystems, Brain Science Institute, Korea Institute of Science and Technology (KIST), Seoul, Korea

[2]Department of Chemical & Biological Engineering, Korea University, Seoul, Korea

[3]Center for Imaging Media Research, Robot & Media Institute, KIST, Seoul, Korea

[4]Department of Applied Chemistry, The Institute of Natural Science, College of Applied Science, Kyung Hee University, Seoul, Korea

*e-mail: sangk@kist.re.kr

S1. Supplementary methods

Target specific forward primers in this paper

miR-9-3p: 5'-ATA AAG CTA GAT AAC CGA AAG T-3'

miR-219-5p: 5'-TGA TTG TCC AAA CGC AAT TCT-3'

miR-16-5p: 5'-TAG CAG CAC GTA AAT ATT GGC G-3'

miR-132-5p: 5'-ACC GTG GCT TTC GAT TGT TAC T-3'

miR-1306-5p: 5'-CCA CCT CCC CTG CAA ACG TCC A-3'

miR-342-3p: 5'-TCT CAC ACA GAA ATC GCA CCC GT-3'

miR-18b-5p: 5'-TAA GGT GCA TCT AGT GCA GTT AG-3'

miR-30e-5p: 5'-TGT AAA CAT CCT TGA CTG GAA G-3'

miR-143-3p: 5'-TGA GAT GA GCA CTG TAG CTC-3'

miR-424-5p: 5’-CAG CAG CAA TTC ATG TTT TGA A-3’

U6 snRNA: 5’-TGG CCC CTG CGC AAG GAT G-3’

Melting curve analysis

Melting curve analysis was performed using a thermal cycler (Cantis, Seoul, Korea) and CFX Connect™ Real-Time PCR (BioRad, Hercules, CA). The melting curves of the PCR amplicons in each LEM-PIN were obtained by increasing the temperature at the rate of 10 ℃/min from 65° C to 95° C. In the thermal cycler, the fluorescent images were obtained at every 0.5° C and their intensities were measured and analyzed with NIH ImageJ software (available at http://rsb.info.nih.gov/ij/).

S2. Procedure for fabrication of LEMs

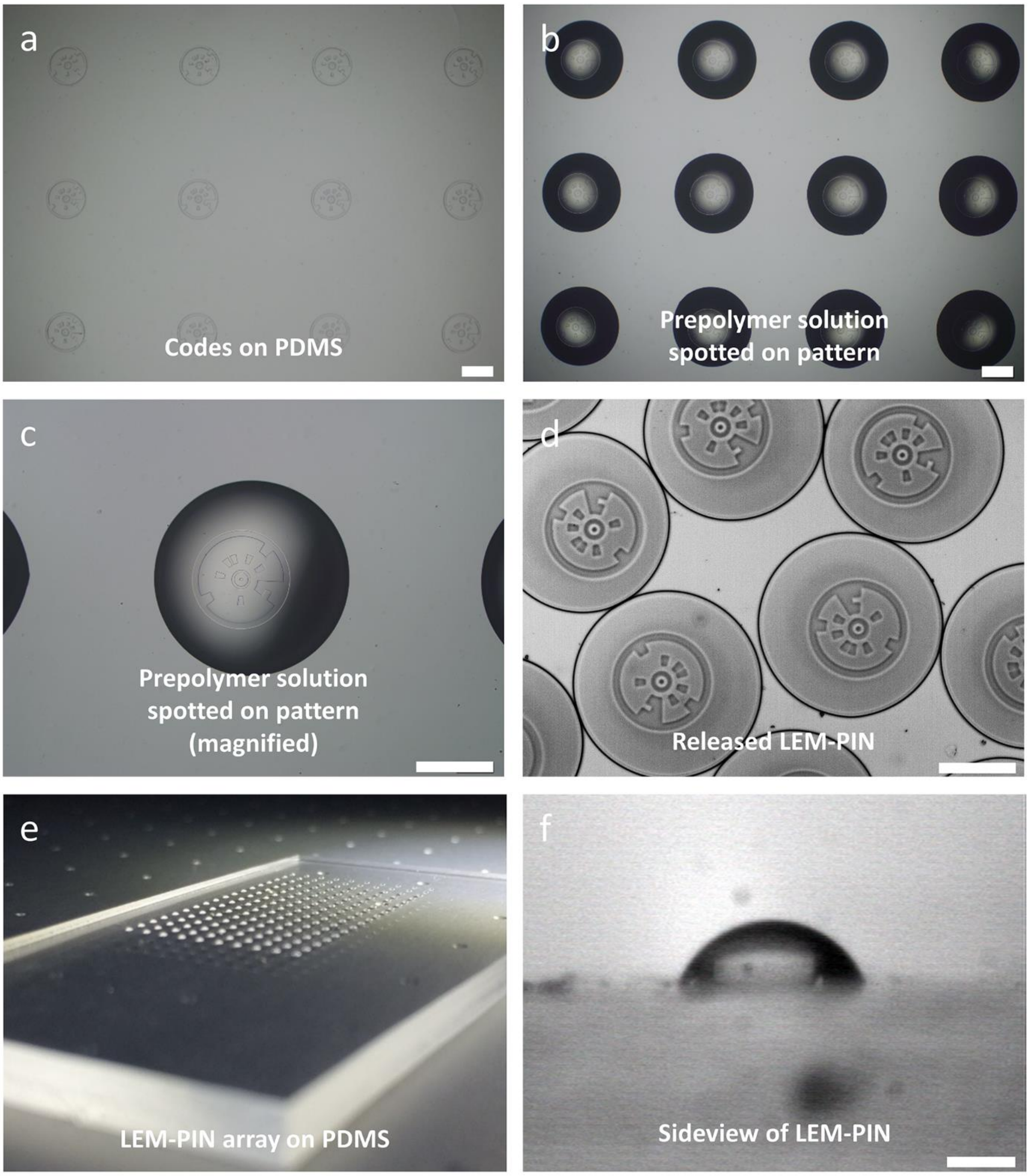

Figure S1 Procedure of LEM-PIN fabrication through microjetting system. a, the 5-μm deep pattern array on PDMS. **b,** LEM-PIN array after jetting and curing on patterns. **c,** Magnified image of LEM-PINs on pattern. **d,** Released and suspended LEM-PINs. **e,** LEM-PIN array on PDMS. **f,** Sideview of a LEM-PIN. All scale bars indicate 200 μm

S3. Various patterns in LEMs

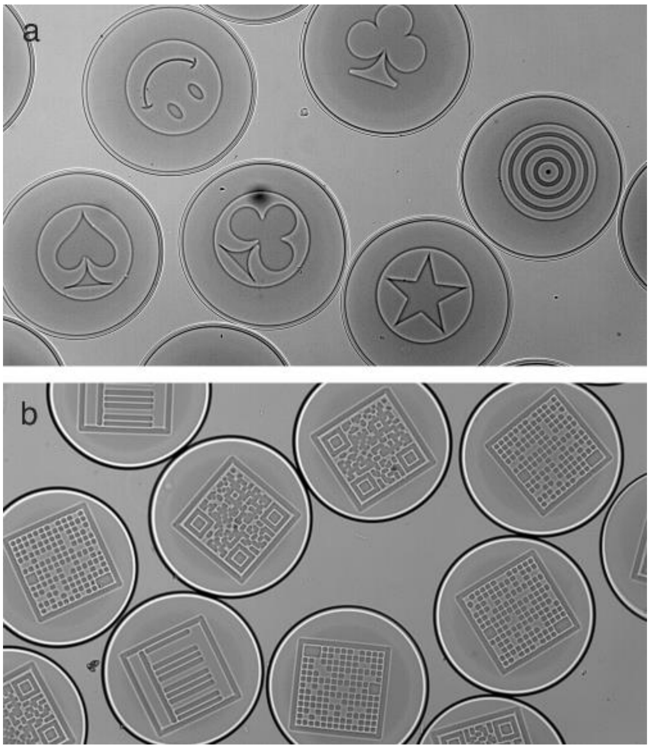

Figure S2 Various codes on LEM-PINs., **a,** various patterns achievable with our system. **b,** 2-D codes including dot, bar, and QR codes.

Figure S2 shows LEM-PINs containing various patterns. Since the patterns on mold were fabricated by photolithography, there was no geometic limitation such as island patterns in concentric rings which were impossible or difficult to achieve in conventional microfluidic system for hydrogel particle generation. [3] Furthermore, the smallest feature size which was achieved in this study was 5 μm in width, which led to extremely high encoding capacity according to diverse coding strategies.

S4. Control of particle size

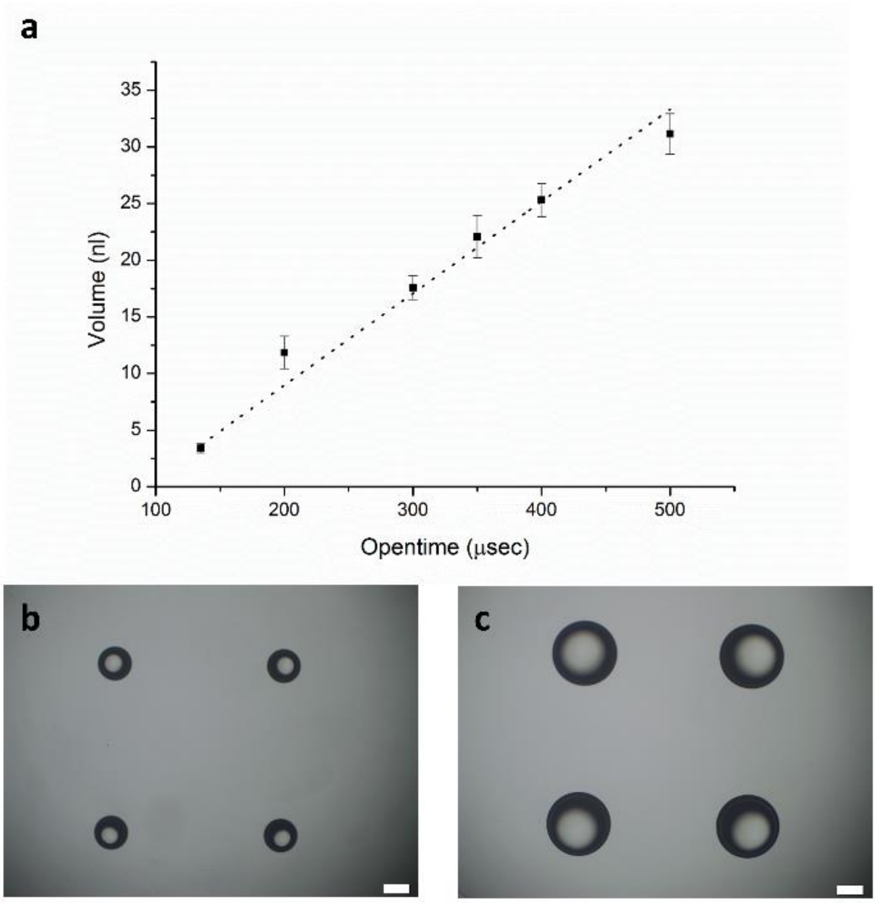

Figure S3 Droplet volume as a function of opentime of solenoid valve when spotting. a, Droplet volume is proportional to the opentime of solenoid valve. **b,** 3 nl droplet array with opentime of 130 μsec. **c,** 25 nl droplet array with opentime of 400 μsec (scale bar= 200 μm).

The opentime of the solenoid valve was modulated to change the dimension of the particles. As increasing the opentime of the solenoid valve, the volume of the particles became linearly larger ranging from 3 nl to 30 nl. The dimension uniformity was quite good with the variation less than 10 % even in the smallest particles.

S5. Decoding of ringcode on LEM-PINs

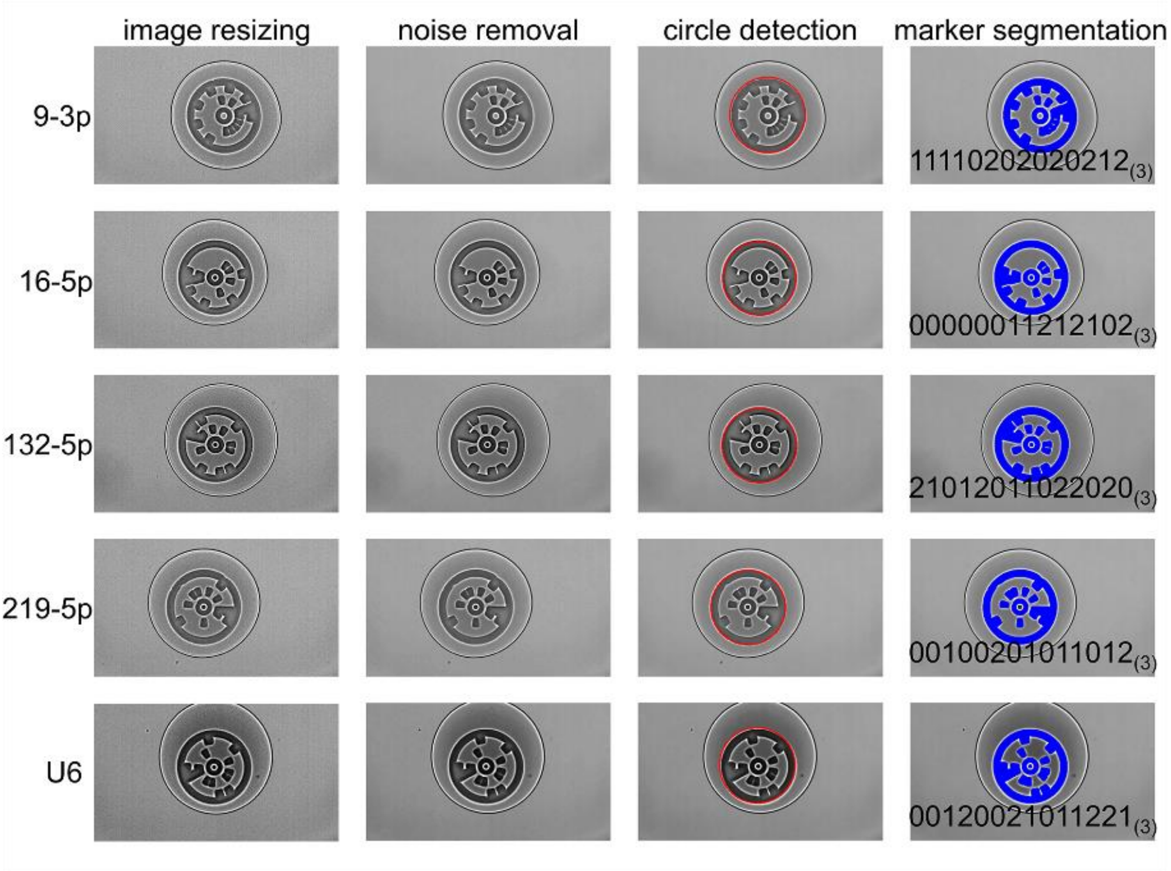

Figure S4 Code extraction process from the images.

The non-local means filter [1] is first applied to reduce the noises in the image while preserving fine details and sharp edges. The circle that enclose the shotcode is then detected using the Hough transform [2] under the assumption that the radius of the shotcode in the image is known according to the image resolution. Finally we use the GrabCut algorithm [3] to segment the pattern, the dark region, inside of the detected circle. Here the pixels around the inside border of the circle are used to model the color distribution of the pattern. The shotcode can then be easily decoded once this pattern is segmented out from the image.

S6. Optimization of primer concentration in PIN

Table S1 Ct values for different primer concentration crosslinked in PIN.

Final concentration of primer in PIN (fmol/nl)	Mean Ct value (triplicate)
**200**	19.44
**100**	19.33
**50**	19.31
**25**	19.33
**10**	19.5
**5**	19.41
**2.5**	21.9
**1**	34.15

In order to see the effect of primer amount existence in PIN during PCR, the serially halved amounts of primer from 200 fmol/nl as a final concentration of primer in PIN were tested through amplification with same concentration of template. As a result, the Ct values showed no significant change by the amount of primer even though lowering to 5 fmol/nl. However, the primer concentration less than 5 fmol/nl led to upshift of Ct values which might be resulted from the lack of the primers to be reacted. For stable experiments in this study, 100 fmol/nl was chosen for final concentration of primer in PIN.

S7. Rinse effect

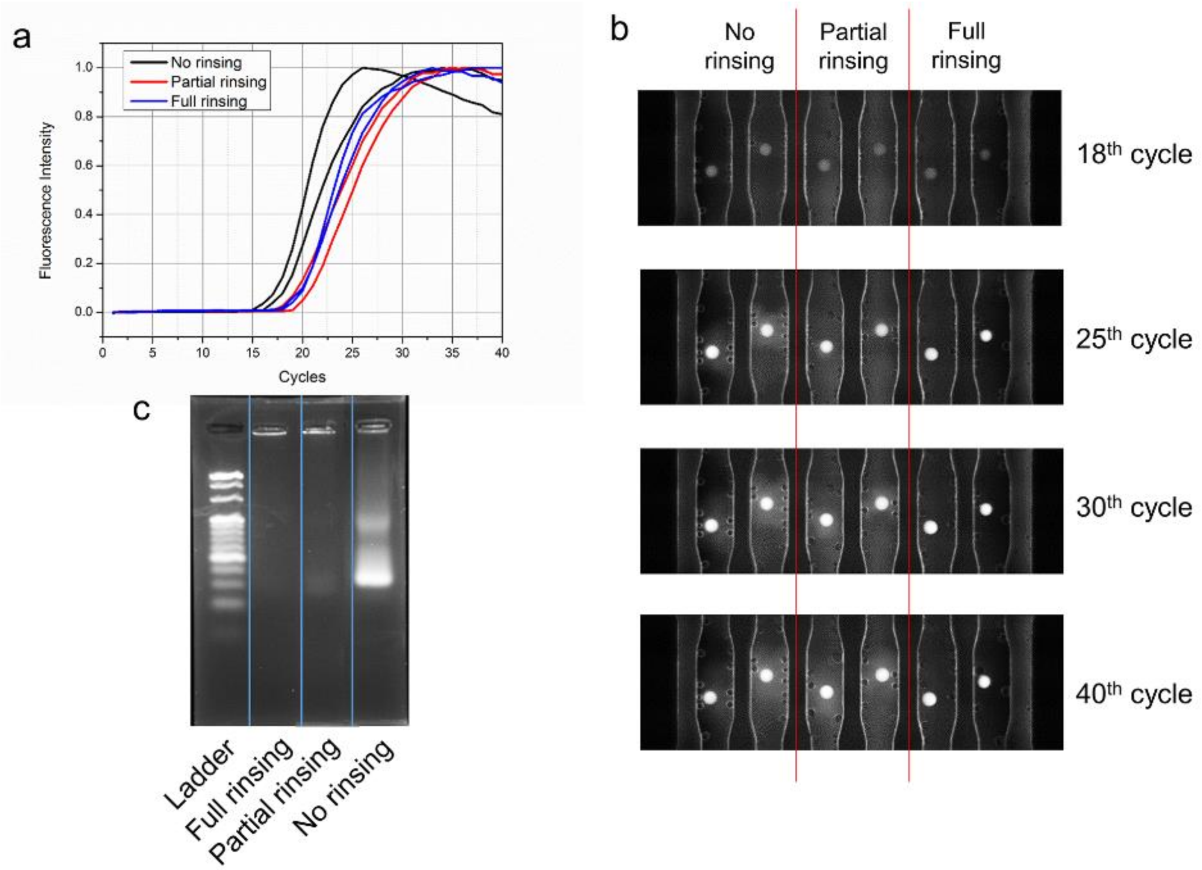

Figure S5 qPCR graphs and images according to rinsing protocols. a, qPCR graphs of the no, partially, or fully rinsed particles. **b,** PCR channel images of the particles made with different rinsing protocols. Insufficient rinsing brought about the varying Ct values (black and read lines in **a**) and dim fluorescence around the particles (channel #1 to #4 in **b**). On the contrary, fully rinsed particles showed consistent graphs (blue lines in **a**) and clear surroundings (channel #5 & #6 in **b**). **c,** In gel electrophoresis, fully rinsed solution showed no band, whereas the others showed bands indicating amplicons generated from free unbound primers.

The rinsing after curing is essential to remove the unbound primers as well as porogen in the particles. The effect of rinsing after curing the particles was described in Fig. S5. Three cases were tested and which were no, partially, and fully rinsed particles. Partial or full rinsing means

the repetition of vortex, centrifugation, and buffer change two or five times, respectively. As a result, there were dim fluorescence around the particles in no and partially rinsed cases, while no fluorescence around fully rinsed particles at all. This was consistent in gel electrophoresis of the surrounding solution of the particles after PCR. There was no band in fully rinsed particles, whereas the others had band indicating amplicons resulted from unbound primers remained in the particles. Based on these experiments, we found that the particles should be rinsed fully in order to confine the fluorescence and eliminate the influence to other particles resulted from the amplification only in the particles.

S8. Comparison between solution-phase and particle-based PCR

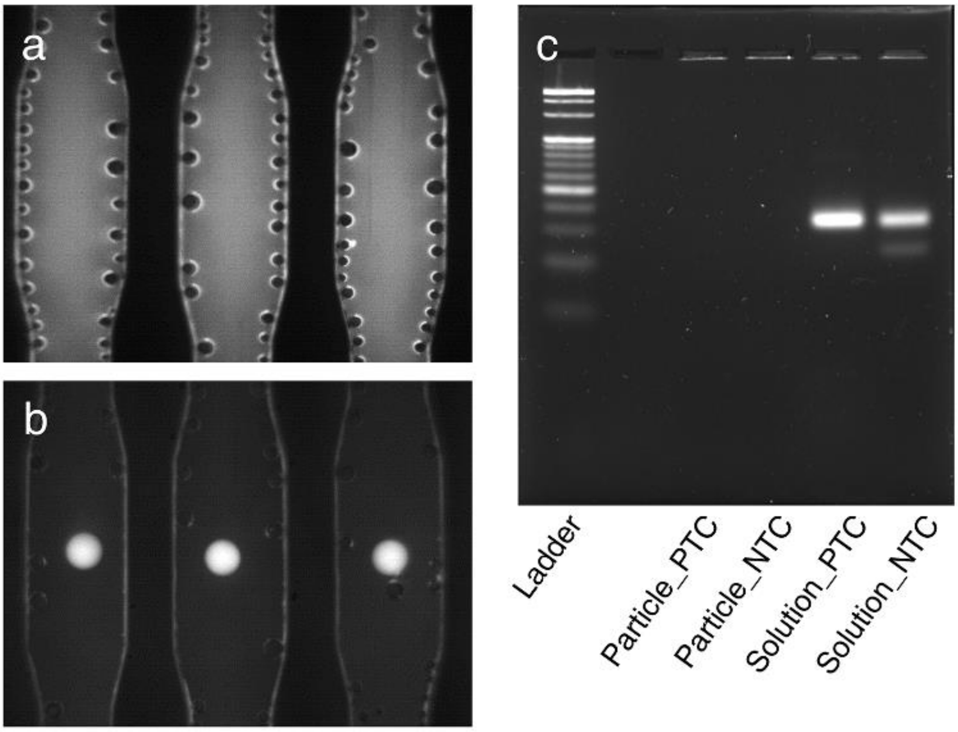

Figure S6 PCR channel and gel electrophoresis after solution-phase and particle-based PCR. a, PCR channel after solution PCR. **b,** PCR channel after particle PCR. **c,** Gel electrophoresis after solution and particle PCRs for PTC and NTC

S9. Quantitative resolution

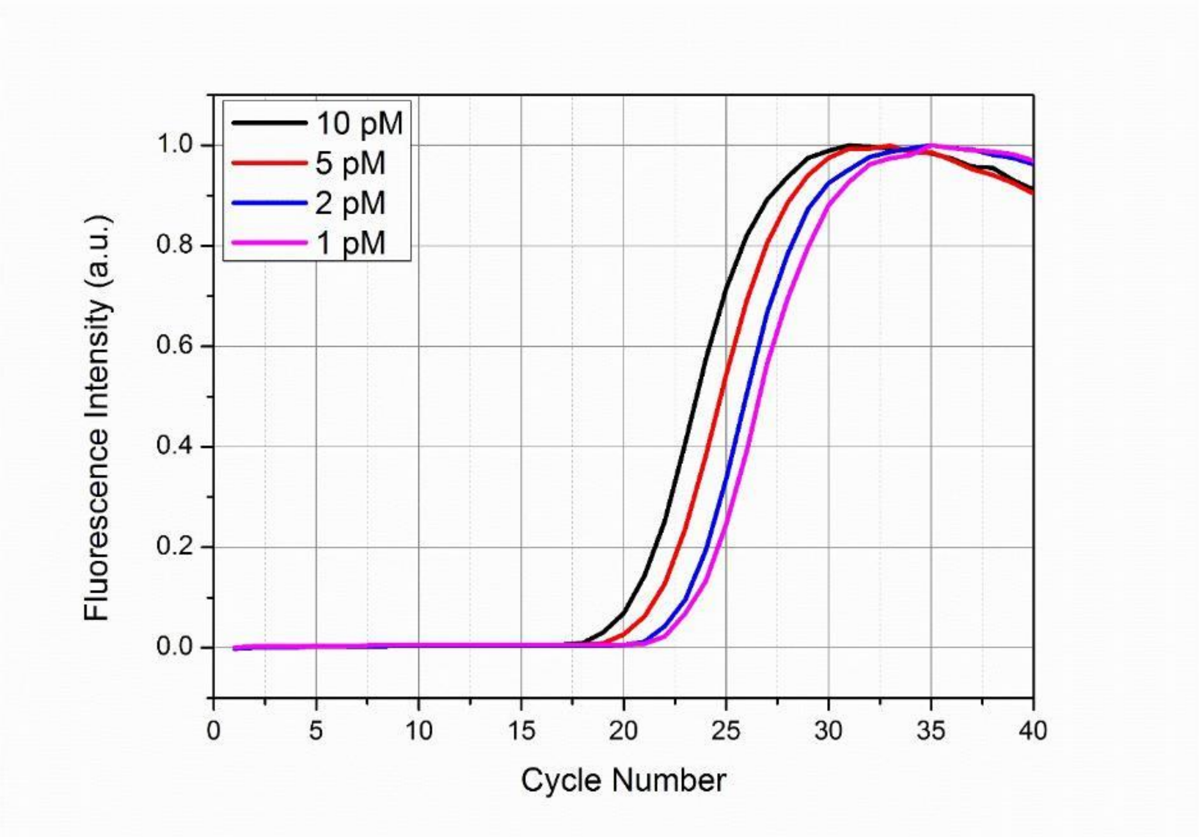

Figure S7 qPCR results with small variation of template concentrations. The data were averaged from triplicate assays in each concentration. Each line showed almost difference of one in Ct value showing halved amount of the template.

For the precisely quantitative analysis of the miRNAs, small variation of the template concentration should be distinguishable in qPCR. The PIN-based qPCRs with small difference in concentration of the template were carried out to see the quantitative resolution. Figure S7 Shows that the double difference in concentration can be clearly distinguished.

S10. Uniformity of performance of PINs in PCR

Table S2 Uniformity of Ct values of particles located in different PCR channels.

Channel	#1	#2	#3	#4	#5	#6	#7	#8	#9	#10	Avg.	Std. dev.
Ct value	19.02	19.09	19.24	19.37	19.09	19.57	19.59	19.7	19.96	20.04	19.47	0.36

Table S3 Uniformity of Ct values of particles in single PCR channel

Particle	#1	#2	#3	#4	#5	#6	#7	#8	#9	#10	#11	Avg.	Std. dev.
Ct value	19.70	19.12	19.37	20.00	18.88	18.75	20.00	20.15	19.37	19.70	19.3	19.49	0.47

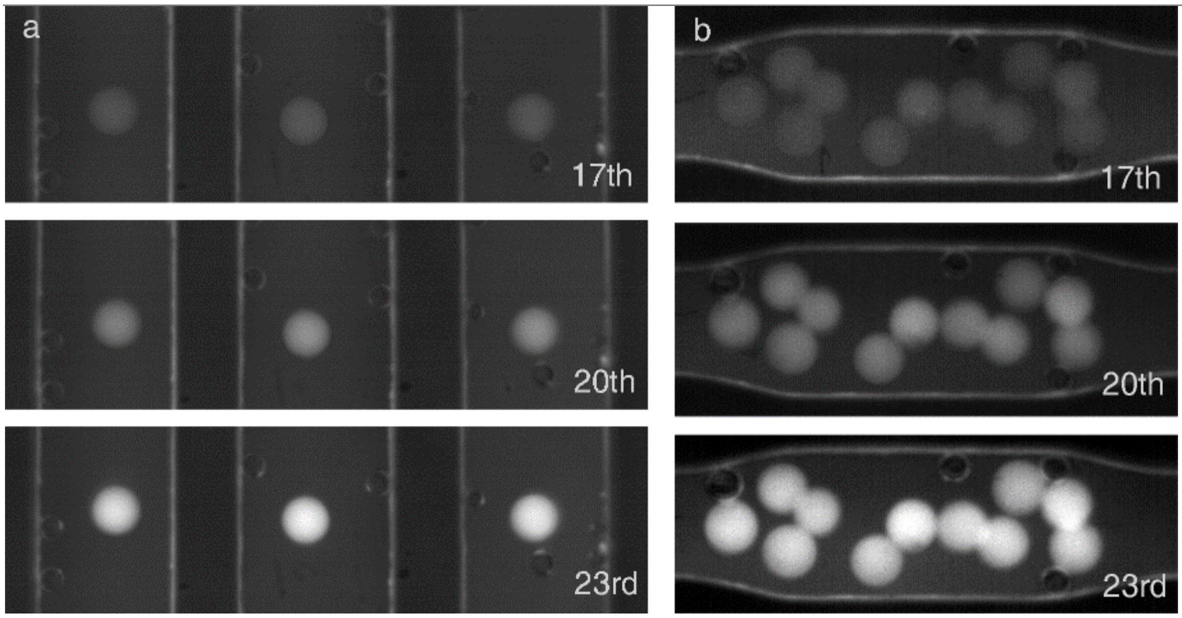

Figure S8 Uniform performance of the particles for qPCR. a, Time lapse snapshots for three particles located in three different channels. **b,** Time lapse snapshots for 11 particles located in single channel.

In order to verify uniformity of particle-based qPCR, we carried out the qPCRs on different channels or many particles in single channel. According to the amplification of the same sample in 10 separate wells, the results showed juxtaposed Ct values at 19.47 with a standard deviation (STD) of 0.36. In addition, the amplification with 11 particles in a single well recorded identical Ct values of 19.49 with a STD of 0.47. Especially uniform Ct value of many particles in single channel is because the amplification was proceeded only with the cDNAs diffused into the particle initially and in- or out-diffusion of the cDNAs was not effective during the

amplification. Thus, the amplification efficiency based on the particle-based qPCR is independent on the number of the particles in the channel.

S11. Multiplex qPCR

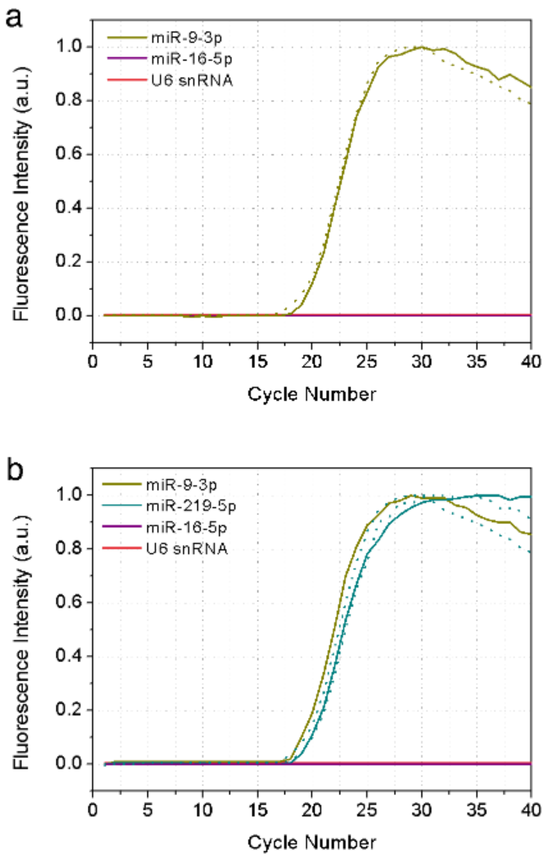

Figure S9 Multiplex qPCR. a, One target was introduced with three different LEM-PINs. Only target-specific LEM-PIN showed the amplification result and it was almost indentical to singleplex result. **b,** Two targets were inserted with four different LEM-PINs. As a result, both of them showed the identical graphs to each singleplex.

S12. Storage stability

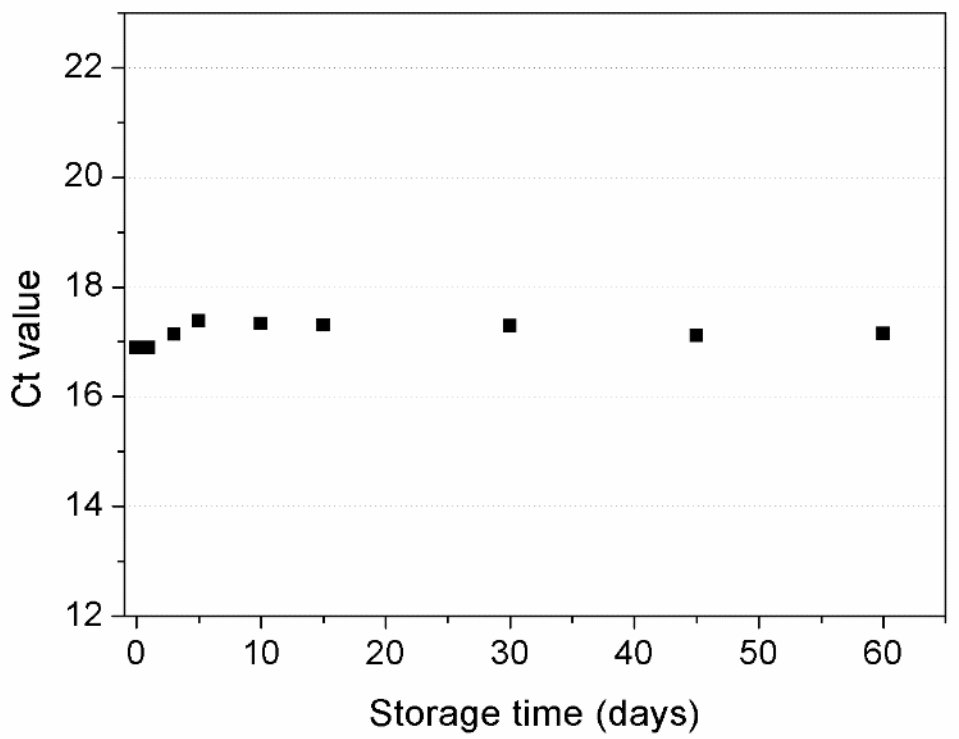

Figure S10 Storage stability of LEM-PIN. The Ct values were consistent regardless of storage time up to two months.

To extend the utility of the LEM-PIN-based qPCR, they should be stored for a long time after massive production. The particles which were stored in -20 ℃ for different periods were used to see the storage stability through the amplification with the identical template concentration. As a result, the Ct values of the particles tested here were uniform regardless of the storage time. Based on this observation, we can claim that the structure of hydrogel particles is stable and cross-linked primers are not degraded for a long time so the particles for qPCR can be stored until before using them.

S13. Melting curve analysis

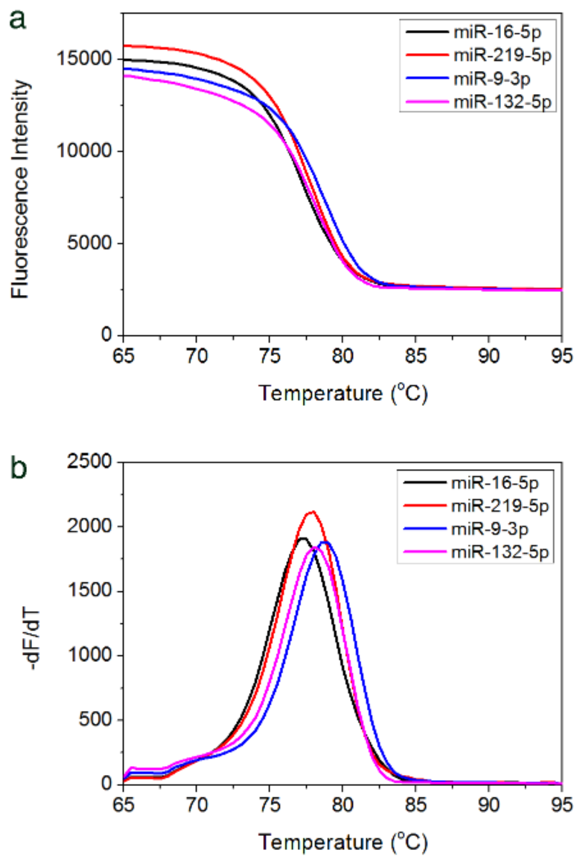

Figure S11 Melting curve analyses of LEM-PINs. a, four different melting curves for each of the four particles. **b,** derived melting cutves. The fluorescence intensities for four different particles were measured by increasing the temperature at the same time. Figure S11b showed clear single peaks for each template.

S14. Detailed analyses of qPCR with EV miRNA

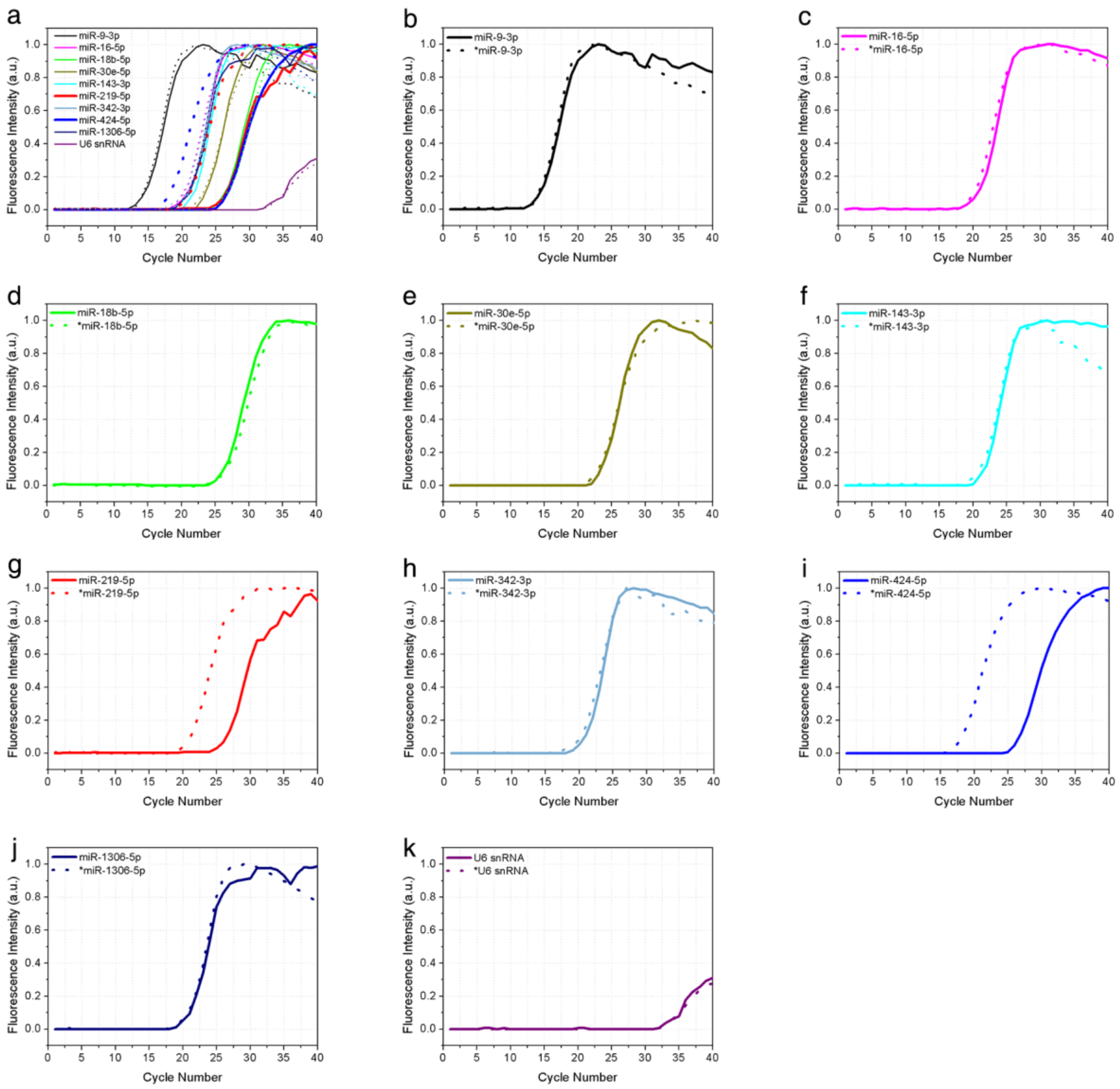

Figure S1 For practical application, the miRNAs of EVs from K562 cell line were analyzed by our protocol. a, the qPCR graphs for control (solid lines) and spiked-in (dot lines) EV samples with 10 kinds of LEM-PINs **b-k,** detailed comparison data for each miRNA. The Ct values for miR-219-5p and miR-424-5p which were spiked in were only downshifted due to increased template amount.

S15. Supplementary movie

The fluorescent snapshots recorded at every cycle of multiplex qPCR with 5 different PIN-LEMs (132-5p, U6 snRNA, 219-5p, 16-5p and 9-3p) shown in Figure 3c were merged in a single movie file. As amplification had been progressed, each PIN-LEM was gradually brightened in order of the amount of miRNAs introduced into the reaction.

References

[1] Buades A., Coll B. & Morel J. A non-local algorithm for image denoising. in *Proc. Int. Conf. Computer Vision and Pattern Recognition (CVPR)* **2,** 60–65 (2005).

[2] Yuen H. K., Princen J., Illingworth J. & Kittler J. Comparative study of Hough transform methods for circle finding. *Image Vision Comput.* **81,** 71–77 (1990).

[3] Rother C., Kolmogorov V. & Blake A. GrabCut: Interactive foreground extraction using iterated graph cuts. *ACM Trans. Graph.* **23,** 309–314 (2004).
